# 2,2-Dimethyl-*N*-(4-methyl­pyridin-2-yl)propanamide

**DOI:** 10.1107/S1600536814003729

**Published:** 2014-02-26

**Authors:** Gamal A. El-Hiti, Keith Smith, Asim A. Balakit, Amany S. Hegazy, Benson M. Kariuki

**Affiliations:** a Department of Optometry, College of Applied Medical Sciences, King Saud University, PO Box 10219, Riyadh 11433, Saudi Arabia; b School of Chemistry, Cardiff University, Main Building, Park Place, Cardiff CF10 3AT, Wales; c Department of Chemistry, College of Science for Women, University of Babylon, Babylon, Iraq

## Abstract

In the title compound, C_11_H_16_N_2_O, the dihedral angle between the mean plane of the 4-methypyridine group and the plane of the amide link is 16.7 (1)°, and there is a short intra­molecular C—H⋯O contact. Hydrogen bonding (N—H⋯O) between amide groups forms chains parallel to the *b* axis. Pairs of methyl­pyridine groups from mol­ecules in adjacent chains are parallel but there is minimal π–π inter­action.

## Related literature   

For biological applications of related compounds, see: de Candia *et al.* (2013 ▶); Thorat *et al.* (2013 ▶); Abdel-Megeed *et al.* (2012 ▶). For convenient routes for modifying pyridine derivatives, see: Smith *et al.* (2013 ▶); Smith *et al.* (2012 ▶); El-Hiti (2003 ▶); Joule & Mills (2000 ▶); Smith *et al.* (1994 ▶, 1995 ▶, 1999 ▶); Turner (1983 ▶). For the X-ray structures of related compounds, see: Mazik & Sicking (2004 ▶); Mazik *et al.* (2004 ▶); Hodorowicz *et al.* (2007 ▶); Koch *et al.* (2008 ▶); Liang *et al.* (2008 ▶); Seidler *et al.* (2011 ▶).
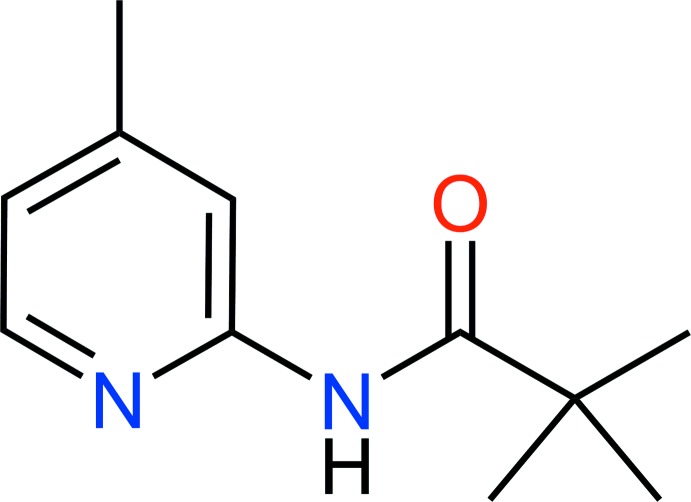


## Experimental   

### 

#### Crystal data  

C_11_H_16_N_2_O
*M* *_r_* = 192.26
Orthorhombic, 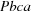

*a* = 10.7954 (3) Å
*b* = 10.1809 (2) Å
*c* = 20.8390 (5) Å
*V* = 2290.35 (10) Å^3^
*Z* = 8
Cu *K*α radiation
μ = 0.58 mm^−1^
*T* = 296 K
0.27 × 0.19 × 0.14 mm

#### Data collection  

Agilent SuperNova (Dual, Cu at zero, Atlas) diffractometer
Absorption correction: gaussian (*CrysAlis PRO*; Agilent, 2014 ▶) *T*_min_ = 0.930, *T*_max_ = 0.957
5219 measured reflections
2253 independent reflections
1808 reflections with *I* > 2σ(*I*)
*R*_int_ = 0.017

#### Refinement  

*R*[*F* ^2^ > 2σ(*F* ^2^)] = 0.046
*wR*(*F* ^2^) = 0.154
*S* = 1.08
2253 reflections
132 parameters
H-atom parameters constrained
Δρ_max_ = 0.16 e Å^−3^
Δρ_min_ = −0.14 e Å^−3^

### 

Data collection: *CrysAlis PRO* (Agilent, 2014 ▶); cell refinement: *CrysAlis PRO*; data reduction: *CrysAlis PRO*; program(s) used to solve structure: *SHELXS2013* (Sheldrick, 2008 ▶); program(s) used to refine structure: *SHELXL2013* (Sheldrick, 2008 ▶); molecular graphics: *ORTEP-3 for Windows* (Farrugia, 2012 ▶); software used to prepare material for publication: *WinGX* (Farrugia, 2012 ▶) and *CHEMDRAW Ultra* (CambridgeSoft, 2001 ▶).

## Supplementary Material

Crystal structure: contains datablock(s) I, New_Global_Publ_Block. DOI: 10.1107/S1600536814003729/mw2120sup1.cif

Structure factors: contains datablock(s) I. DOI: 10.1107/S1600536814003729/mw2120Isup2.hkl

Supporting information file. DOI: 10.1107/S1600536814003729/mw2120Isup3.cml

CCDC reference:

Additional supporting information:  crystallographic information; 3D view; checkCIF report

## Figures and Tables

**Table 1:** Hydrogen-bond geometry (Å, °)

*D*—H⋯*A*	*D*—H	H⋯*A*	*D*⋯*A*	*D*—H⋯*A*
N2—H2*A*⋯O1^i^	0.86	2.22	3.0644 (17)	168
C5—H5⋯O1	0.93	2.28	2.842 (2)	118

Symmetry code: (i) 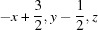.

**Fig. 1.**
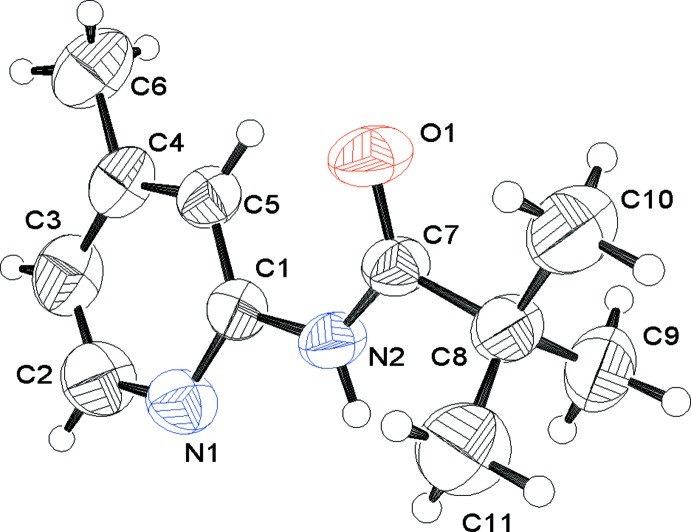
A molecule showing atom labels and 50% probability displacement ellipsoids for non-H atoms.

**Fig. 2.**
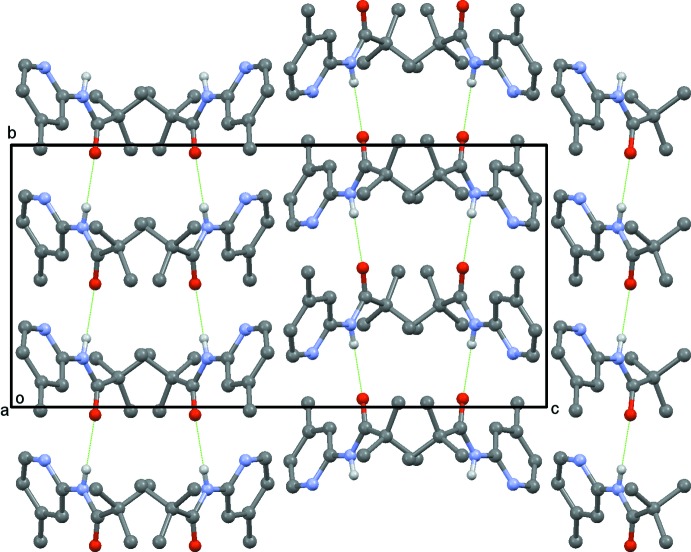
Crystal structure packing showing NH..O hydrogen bonds as green dotted lines with the rest of the hydrogen atoms omitted for clarity.

## supplementary crystallographic information

### 2. Structural commentary

Synthetic and naturally occurring pyridine derivatives have a broad range of biological activities (Thorat *et al.*, 2013) including anti­cancer and anti­microbial (Abdel-Megeed *et al.*, 2012) and anti­coagulant (de Candia *et al.*, 2013) properties. Hence, pyridine derivatives are important compounds (Joule and Mills, 2000) and some synthetic approaches involve li­thia­tion of 2-acyl­amino­pyridines (Smith *et al.*, 1995; Turner, 1983). The structures of a number of 2-acyl­amino­pyridines have been determined (Mazik & Sicking, 2004; Mazik *et al.*, 2004; Hodorowicz *et al.*, 2007; Koch *et al.*, 2008; Liang *et al.*, 2008; Seidler *et al.*, 2011). During research focused on new synthetic routes towards novel substituted pyridine derivatives (Smith *et al.*, 1994; Smith *et al.*, 1995; Smith *et al.*, 1999; El-Hiti, 2003; Smith *et al.*, 2012; Smith *et al.*, 2013) we have synthesized the title compound in high yield. In the 4-methyl-2-pivaloyl­amino­pyridine molecule (Figure 1), the least squares plane through the 4-methypyridine group makes a dihedral angle of 16.7 (1)° with the plane through the amide link and a short intra­molecular C5—H5···O1 contact is observed (Table 1). In the crystal structure (Figure 2) N—H···O hydrogen bonding between amide groups forms chains parallel to the *b* axis. Pairs of methyl-pyridine groups in molecules from adjacent chains are parallel but there is minimal π-π inter­action. The ring nitro­gen is not involved in strong hydrogen bonding.

### 5. Synthesis and crystallization

To a cooled solution (0 °C) of 2-amino-4-methyl­pyridine (5.41 g, 50.0 mmol) and tri­ethyl­amine (10 ml) in di­chloro­methane (DCM, 80 ml) pivaloyl chloride (6.63 g, 55.0 mmol) was slowly added in a drop-wise manner over 10 min. The reaction mixture was stirred at 0 °C for an extra 1 h. The mixture was poured into H_2_O (100 ml) and the organic layer was separated, washed with H_2_O (2 × 50 ml), dried (MgSO_4_) and evaporated under reduced pressure to remove the solvent. The solid obtained was purified by crystallization from Et_2_O–hexane (2:1) to give 4-methyl-2-pivaloyl­amino­pyridine (9.04 g, 47.0 mmol; 94%) as colourless crystals, m.p. 103–104 °C [lit. 96–98 °C (hexane); Turner (1983)]. ^1^H NMR (500 MHz, CDCl_3_, *δ*, p.p.m.) 8.11–8.10 (br, 2 H, H-3 and H-6), 8.05 (br, exch., 1 H, NH), 6.85 (m, 1 H, H-5), 2.34 (s, 3 H, CH_3_), 1.31 [s, 9 H, C(CH_3_)_3_]. ^13^CNMR (125 MHz, CDCl_3_, *δ*, p.p.m.) 177.2 (s, C=O), 151.5 (s, C-4), 149.9 (s, C-2), 147.2 (d, C-6), 120.9 (d, C-5), 114.5 (d, C-3), 39.8 [s, *C*(CH_3_)_3_], 27.5 [q, C(*C*H_3_)_3_]), 21.4 (q, CH_3_). EI^+^–MS (*m/z*, %): 192 (*M*^+^, 43), 177 (5), 149 (11), 135 (25), 108 (100), 92 (15), 81 (15), 57 (25). HRMS (EI^+^): Calculated for C_11_H_16_N_2_O [*M*] 192.1263; found, 192.1260.

### 6. Refinement

Crystal data, data collection and structure refinement details are summarized in Table 1. H atoms were positioned geometrically and refined using a riding model with *U*_iso_(H) = 1.2 times *U*_eq_ for the atom they are bonded to except for the methyl groups where 1.5 times *U*_eq_ was used with free rotation about the C—C bond.

### Crystal data

**Table:**

C_11_H_16_N_2_O	*D*_x_ = 1.115 Mg m^−^^3^
*M**_r_* = 192.26	Cu *K*α radiation, λ = 1.54184 Å
Orthorhombic, *P**b**c**a*	Cell parameters from 1808 reflections
*a* = 10.7954 (3) Å	θ = 4.2–74.0°
*b* = 10.1809 (2) Å	µ = 0.58 mm^−^^1^
*c* = 20.8390 (5) Å	*T* = 296 K
*V* = 2290.35 (10) Å^3^	Block, colourless
*Z* = 8	0.27 × 0.19 × 0.14 mm
*F*(000) = 832	

### Data collection

**Table:**

Agilent SuperNova (Dual, Cu at zero, Atlas) diffractometer	1808 reflections with *I* > 2σ(*I*)
Radiation source: sealed X-ray tube	*R*_int_ = 0.017
ω scans	θ_max_ = 74.0°, θ_min_ = 4.2°
Absorption correction: gaussian (*CrysAlis PRO*; Agilent, 2014)	*h* = −7→13
*T*_min_ = 0.930, *T*_max_ = 0.957	*k* = −12→8
5219 measured reflections	*l* = −25→20
2253 independent reflections	

### Refinement

**Table:**

Refinement on *F*^2^	Hydrogen site location: inferred from neighbouring sites
Least-squares matrix: full	H-atom parameters constrained
*R*[*F*^2^ > 2σ(*F*^2^)] = 0.046	*w* = 1/[σ^2^(*F*_o_^2^) + (0.0734*P*)^2^ + 0.4299*P*] where *P* = (*F*_o_^2^ + 2*F*_c_^2^)/3
*wR*(*F*^2^) = 0.154	(Δ/σ)_max_ < 0.001
*S* = 1.08	Δρ_max_ = 0.16 e Å^−^^3^
2253 reflections	Δρ_min_ = −0.14 e Å^−^^3^
132 parameters	Extinction correction: *SHELXL2013* (Sheldrick, 2008), Fc^*^=kFc[1+0.001xFc^2^λ^3^/sin(2θ)]^-1/4^
0 restraints	Extinction coefficient: 0.0037 (5)

### Special details

**Table:**

Experimental. Absorption correction: CrysAlisPro (Agilent, 2014): Numerical absorption correction based on Gaussian integration over a multifaceted crystal model. Empirical absorption correction using spherical harmonics, implemented in SCALE3 ABSPACK scaling algorithm.
Geometry. All e.s.d.'s (except the e.s.d. in the dihedral angle between two l.s. planes) are estimated using the full covariance matrix. The cell e.s.d.'s are taken into account individually in the estimation of e.s.d.'s in distances, angles and torsion angles; correlations between e.s.d.'s in cell parameters are only used when they are defined by crystal symmetry. An approximate (isotropic) treatment of cell e.s.d.'s is used for estimating e.s.d.'s involving l.s. planes.

### Fractional atomic coordinates and isotropic or equivalent isotropic displacement parameters (Å^2^)

**Table:**

	*x*	*y*	*z*	*U*_iso_*/*U*_eq_	
C1	0.90730 (14)	0.81479 (14)	0.59591 (7)	0.0489 (4)	
C2	1.0228 (2)	0.6963 (2)	0.52600 (11)	0.0816 (6)	
H2	1.0345	0.6201	0.5021	0.098*	
C3	1.1102 (2)	0.7922 (2)	0.52141 (11)	0.0792 (6)	
H3	1.1786	0.7811	0.4948	0.095*	
C4	1.09637 (16)	0.90571 (19)	0.55651 (9)	0.0632 (5)	
C5	0.99149 (14)	0.91744 (16)	0.59451 (8)	0.0552 (4)	
H5	0.9779	0.9929	0.6186	0.066*	
C6	1.1912 (2)	1.0138 (3)	0.55444 (12)	0.0922 (7)	
H6A	1.2409	1.0107	0.5926	0.138*	
H6B	1.1499	1.0972	0.5520	0.138*	
H6C	1.2431	1.0025	0.5175	0.138*	
C7	0.74226 (15)	0.91931 (14)	0.66136 (8)	0.0515 (4)	
C8	0.62472 (16)	0.88824 (16)	0.69967 (9)	0.0606 (5)	
C9	0.6598 (2)	0.8017 (2)	0.75696 (11)	0.0851 (7)	
H9A	0.7235	0.8442	0.7815	0.128*	
H9B	0.6895	0.7185	0.7418	0.128*	
H9C	0.5882	0.7883	0.7835	0.128*	
C10	0.5677 (2)	1.0158 (2)	0.72312 (13)	0.0997 (9)	
H10A	0.6251	1.0600	0.7509	0.149*	
H10B	0.4929	0.9970	0.7463	0.149*	
H10C	0.5490	1.0709	0.6870	0.149*	
C11	0.53133 (19)	0.8146 (3)	0.65810 (13)	0.0933 (8)	
H11A	0.4578	0.7976	0.6826	0.140*	
H11B	0.5668	0.7329	0.6442	0.140*	
H11C	0.5107	0.8670	0.6213	0.140*	
N1	0.92138 (14)	0.70437 (14)	0.56251 (8)	0.0654 (4)	
N2	0.79937 (12)	0.81473 (12)	0.63366 (7)	0.0554 (4)	
H2A	0.7654	0.7394	0.6400	0.067*	
O1	0.78283 (12)	1.03049 (11)	0.65664 (7)	0.0699 (4)	

### Atomic displacement parameters (Å^2^)

**Table:**

	*U*^11^	*U*^22^	*U*^33^	*U*^12^	*U*^13^	*U*^23^
C1	0.0475 (8)	0.0469 (8)	0.0523 (8)	0.0056 (6)	0.0003 (6)	0.0012 (6)
C2	0.0788 (13)	0.0735 (12)	0.0924 (14)	0.0131 (10)	0.0224 (11)	−0.0152 (11)
C3	0.0626 (11)	0.0889 (14)	0.0861 (13)	0.0180 (10)	0.0226 (10)	0.0029 (11)
C4	0.0481 (9)	0.0720 (11)	0.0694 (10)	0.0027 (8)	0.0020 (7)	0.0171 (9)
C5	0.0518 (9)	0.0531 (9)	0.0607 (9)	0.0001 (7)	0.0028 (7)	0.0032 (7)
C6	0.0587 (11)	0.1052 (17)	0.1126 (18)	−0.0166 (11)	0.0083 (11)	0.0244 (15)
C7	0.0533 (8)	0.0390 (7)	0.0622 (8)	0.0022 (6)	0.0061 (7)	−0.0009 (6)
C8	0.0592 (10)	0.0478 (8)	0.0749 (10)	0.0016 (7)	0.0190 (8)	−0.0021 (7)
C9	0.0969 (16)	0.0788 (13)	0.0798 (13)	0.0035 (12)	0.0275 (12)	0.0104 (10)
C10	0.1026 (17)	0.0605 (12)	0.136 (2)	0.0135 (11)	0.0640 (16)	−0.0025 (12)
C11	0.0548 (11)	0.1121 (19)	0.1129 (18)	−0.0056 (11)	0.0126 (12)	−0.0159 (15)
N1	0.0646 (9)	0.0546 (8)	0.0770 (9)	0.0056 (7)	0.0107 (7)	−0.0124 (7)
N2	0.0551 (8)	0.0401 (7)	0.0711 (8)	−0.0038 (5)	0.0153 (6)	−0.0040 (6)
O1	0.0673 (8)	0.0389 (6)	0.1034 (10)	−0.0010 (5)	0.0213 (7)	−0.0030 (6)

### Geometric parameters (Å, º)

**Table:**

C1—N1	1.3310 (19)	C7—N2	1.3590 (19)
C1—C5	1.385 (2)	C7—C8	1.532 (2)
C1—N2	1.406 (2)	C8—C10	1.518 (2)
C2—N1	1.336 (3)	C8—C11	1.526 (3)
C2—C3	1.361 (3)	C8—C9	1.531 (3)
C2—H2	0.9300	C9—H9A	0.9600
C3—C4	1.376 (3)	C9—H9B	0.9600
C3—H3	0.9300	C9—H9C	0.9600
C4—C5	1.387 (2)	C10—H10A	0.9600
C4—C6	1.503 (3)	C10—H10B	0.9600
C5—H5	0.9300	C10—H10C	0.9600
C6—H6A	0.9600	C11—H11A	0.9600
C6—H6B	0.9600	C11—H11B	0.9600
C6—H6C	0.9600	C11—H11C	0.9600
C7—O1	1.2177 (18)	N2—H2A	0.8600
			
N1—C1—C5	123.45 (15)	C11—C8—C9	108.85 (18)
N1—C1—N2	112.76 (13)	C10—C8—C7	109.09 (14)
C5—C1—N2	123.78 (14)	C11—C8—C7	110.66 (15)
N1—C2—C3	124.35 (19)	C9—C8—C7	108.69 (15)
N1—C2—H2	117.8	C8—C9—H9A	109.5
C3—C2—H2	117.8	C8—C9—H9B	109.5
C2—C3—C4	119.33 (18)	H9A—C9—H9B	109.5
C2—C3—H3	120.3	C8—C9—H9C	109.5
C4—C3—H3	120.3	H9A—C9—H9C	109.5
C3—C4—C5	117.68 (17)	H9B—C9—H9C	109.5
C3—C4—C6	121.72 (19)	C8—C10—H10A	109.5
C5—C4—C6	120.60 (19)	C8—C10—H10B	109.5
C1—C5—C4	118.87 (16)	H10A—C10—H10B	109.5
C1—C5—H5	120.6	C8—C10—H10C	109.5
C4—C5—H5	120.6	H10A—C10—H10C	109.5
C4—C6—H6A	109.5	H10B—C10—H10C	109.5
C4—C6—H6B	109.5	C8—C11—H11A	109.5
H6A—C6—H6B	109.5	C8—C11—H11B	109.5
C4—C6—H6C	109.5	H11A—C11—H11B	109.5
H6A—C6—H6C	109.5	C8—C11—H11C	109.5
H6B—C6—H6C	109.5	H11A—C11—H11C	109.5
O1—C7—N2	122.06 (15)	H11B—C11—H11C	109.5
O1—C7—C8	122.14 (14)	C1—N1—C2	116.32 (16)
N2—C7—C8	115.80 (13)	C7—N2—C1	127.83 (13)
C10—C8—C11	109.58 (19)	C7—N2—H2A	116.1
C10—C8—C9	109.95 (18)	C1—N2—H2A	116.1

### Hydrogen-bond geometry (Å, º)

**Table:**

*D*—H···*A*	*D*—H	H···*A*	*D*···*A*	*D*—H···*A*
N2—H2*A*···O1^i^	0.86	2.22	3.0644 (17)	168
C5—H5···O1	0.93	2.28	2.842 (2)	118

Symmetry code: (i) −*x*+3/2, *y*−1/2, *z*.
